# Anatomy-dependent lung doses from 3D-conformal breast-cancer radiotherapy

**DOI:** 10.1038/s41598-022-14149-2

**Published:** 2022-06-28

**Authors:** Pavel Kundrát, Hannes Rennau, Julia Remmele, Sabine Sebb, Cristoforo Simonetto, Jan Christian Kaiser, Guido Hildebrandt, Ulrich Wolf, Markus Eidemüller

**Affiliations:** 1 Department of Radiation Sciences, Institute of Radiation Medicine, Helmholtz Zentrum München GmbH - German Research Center for Environmental Health, Ingolstädter Landstr. 1, 85764 Neuherberg, Germany; 2 Department of Radiation Dosimetry, Nuclear Physics Institute of the CAS, Na Truhlářce 39/64, 180 00 Prague 8, Libeň Czech Republic; 3 Department of Radiation Oncology, Universitätsmedizin Rostock Klinik und Poliklinik für Strahlentherapie, MVZ der Universitätsmedizin Rostock am Standort Südstadt gGmbH, Südring 75, 18059 Rostock, Germany; 4 Department of Radiation Oncology, Universität Leipzig Klinik für Strahlentherapie, Stephanstraße 9a, 04103 Leipzig, Germany

**Keywords:** Breast cancer, Radiotherapy

## Abstract

This study aims to identify key anatomic features that govern the individual variability of lung doses from breast-cancer radiotherapy. 3D conformal, intensity-modulated and hybrid techniques with 50.4 Gy whole-breast dose were planned for 128 patients. From their CT images, 17 anatomic measures were assessed and tested as predictors for lung dose-volume characteristics. Tangential techniques yielded mean ipsilateral lung doses in the range of 3–11 Gy. This inter-patient variability was explained to almost 40% by central lung distance, and to almost 60% if this measure was complemented by midplane lung width and maximum heart distance. Also the variability in further dose-volume metrics such as volume fractions receiving 5, 20 or 40 Gy could be largely explained by the anatomy. Multi-field intensity-modulated radiotherapy reduced high-exposed lung volumes, but resulted in higher mean ipsilateral lung doses and larger low-dose burden. Contralateral lung doses ranged from 0.3 to 1 Gy. The results highlight that there are large differences in lung doses among breast-cancer patients. Most of this inter-individual variability can be explained by a few anatomic features. The results will be implemented in a dedicated software tool to provide personalized estimates of long-term health risks related to breast-cancer radiotherapy. The results may also be used to identify favourable as well as problematic anatomies, and serve as a quick quantitative benchmark for individual treatment plans.

## Introduction

Adjuvant radiotherapy reduces both recurrence and mortality rates in breast-cancer patients^1^. Exposures of the lung, which unfortunately cannot be avoided completely, may lead to pneumonitis, lung fibrosis and/or radiation-induced lung cancers^1–3^. These risks generally increase with radiation exposure, and depend on other factors such as the patient’s age, genetic background or tobacco smoking. For instance, typical breast-cancer radiotherapy with a whole-lung dose of 5 Gy administered to a 50-year-old patient was estimated to enhance her risk of death from lung cancer before age 80 years from 0.5 to 0.8% (an absolute increase of 0.3%) for a non-smoker, but from 9.4 to 13.8% (an absolute increase of 4.4%) for a long-term smoker^2^.

Lung doses from breast-cancer radiotherapy differ among alternative irradiation techniques: compared with classical 3D-conformal tangential irradiation, the techniques of intensity-modulated radiotherapy (IMRT) or volumetric modulated arc therapy (VMAT) increase the low-dose spill to the lungs^4^. Inclusion of regional lymph nodes (RNI) also results in higher lung doses^4,5^.

Importantly, lung doses exhibit a high individual variability due to variations in patients’ anatomies. Central lung distance (CLD), i.e. the extent of the lung covered by a tangential field, has previously been suggested as an anatomic measure that correlates with the mean dose and further dose-volume metrics of this organ^6–9^. For instance, for left-sided breast-cancer patients irradiated by 2D tangential techniques^9^, mean dose to the ipsilateral lung (IL) increased by 4.3 Gy per 1 cm increase in CLD, and on average reached 14 Gy.

Contemporary radiotherapy techniques have succeeded in considerably reducing lung doses. In a recent review, mean doses of 9 Gy to IL and 2.2 Gy to the contralateral lung (CL) have been reported^10^. However, their individual variability has not been studied in detail. To address this issue, we present anatomy-dependent lung doses from whole-breast radiotherapy (without RNI) achievable using classical tangential techniques. We show that individual variations in mean lung doses and further dose-volume characteristics are largely covered by a few anatomic measures. The present data may serve as a benchmark on plan quality or as a fast tool needed for estimating person-specific risks before calculating actual treatment plans, as well as for identifying patients with favourable vs. problematic anatomies.

## Materials and methods

### Treatment planning dataset

This work was a part of the German national project PASSOS, which aimed at assessing long-term risks from breast-cancer radiotherapy on a personalized basis^11^. This study includes data from treatment plans for 78 and 50 early-stage female breast-cancer patients (TNM-classification pT1–2, tumour size < 3 cm, pN0, G1–3, R0) from the Departments of Radiation Oncology at University Hospitals Leipzig and Rostock, Germany (hereafter centres 1 and 2). Patients with left-sided tumours represented the majority of the study (45 and 27 patients in centre 1 and 2, respectively), since heart risks are higher for left- than right-sided breast-cancer patients. The treated breast, IL and CL as well as further organs not discussed in this work were contoured manually, using Radiation Therapy Oncology Group (RTOG) consensus definitions for radiation therapy planning^12^.

Treatment plans were generated with 3D-conformal radiotherapy without physical wedges (3DCRT-w), using two opposing tangential fields complemented by about one to four additional asymmetric fields (the number of additional segments depended on individual patient geometry). Compared with techniques involving wedges, this method was shown to improve dose coverage and homogeneity in the PTV as well as to reduce scattered dose to organs at risk^13,14^. In addition, alternatively plans were calculated in parallel. To assess how lung doses are affected by the use of wedges, 3D-conformal radiotherapy with physical wedges (3DCRT + w) was planned for the 78 patients in centre 1. To address potential variation caused by using inverse instead of forward planning, in addition two hybrid techniques were planned for the same patients. In these techniques, the same tangential fields as 3DCRT-w and 3DCRT+w were used to deliver 70–80% of the prescribed whole-breast dose. They were topped up by inversely planned saturating fields, using either flattened (FF) or flattening filter-free (FFF) beams, in both cases without wedges. Finally, to enable comparisons of lung doses from 3DCRT and intensity-modulated radiotherapy (IMRT), multi-field step-and-shoot IMRT was planned for the 50 patients in centre 2; typically 11–15 beams were used.

All plans were generated and approved as if actually delivered in the respective centre. The prescribed whole-breast dose was 50.4 Gy in 28 fractions. In addition to the coverage of the treated breast, all techniques aimed at sparing the heart, ipsilateral lung and contralateral breast. In centre 1, 3DCRT-w, 3DCRT+w and FF were planned for 6 MV photon beams (for 3DCRT-w in combination with 10 MV) from Siemens Primus (Siemens AG, Healthcare, Erlangen, Germany), and FFF was based on 7 MV photons from Siemens Artiste. In centre 2, 3DCRT-w and IMRT used photons from Siemens Oncor Impression Plus. All plans were generated with Oncentra Masterplan treatment planning system (TPS) with dose calculations based on the collapsed cone algorithm. Additional details can be found elsewhere^15–18^.

### Anatomic features

**Figure 1:**
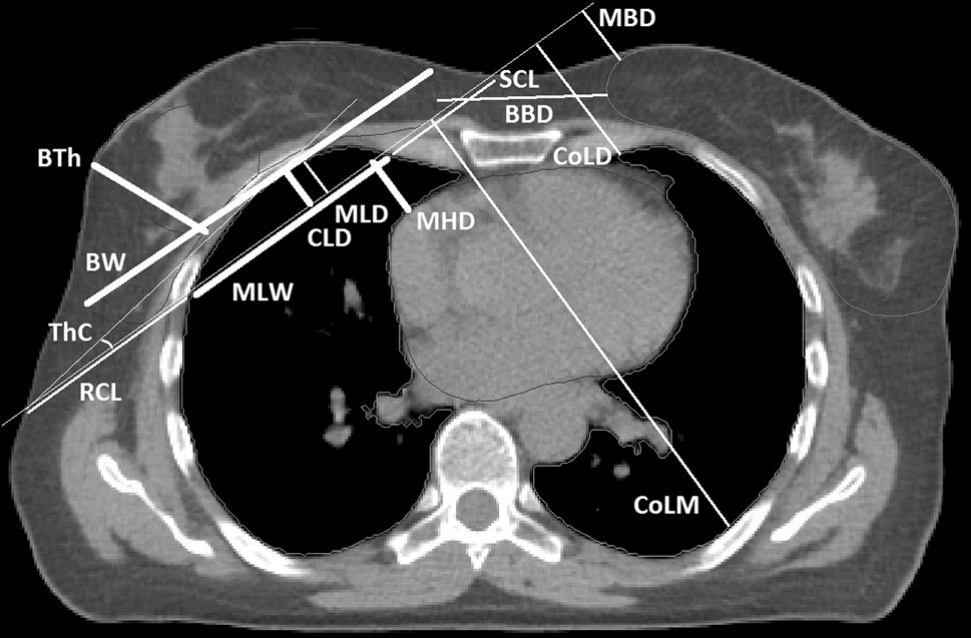
Anatomic measures scored from patients’ CT images. Cranio-caudal measures are not shown.

The PASSOS project aimed at analysing anatomy-dependent dose distributions not only in the lungs but also in other critical organs. To this end, in addition to basic information on tumour laterality and its location in/between breast quadrants, 17 measures reflecting thorax anatomy were assessed from the patient’s CT data within the project^16^. The treated breast was delineated in the CT data, the breast tangent was drawn and anatomic measures were defined as described below and illustrated in Fig. 1.

First, there were measures related to the treated breast itself. Breast width (BW) and breast thickness (BTh) were measured in the breast midplane CT slice. Breast length (BL, not depicted in Fig. 1) was assessed in cranio-caudal direction, and required scrolling through the CT slices.

Then, there were measures that captured IL anatomy relative to the treated breast. Midplane lung width (MLW) was defined as the chord length of the breast tangent through the IL, measured in the breast midplane CT slice. In the same slice, midplane lung distance (MLD) was scored as the extent of IL beyond the breast tangent. Central lung distance (CLD) was defined as the maximum extent of IL beyond the breast tangent over all CT slices; since the maximum may occur in another slice than the midplane one, it holds CLD ≥ MLD. Central lung length (CLL, not shown in Fig. 1) was measured in cranio-caudal direction.

Two parameters were introduced to capture CL anatomy. Contralateral lung distance (CoLD) was measured as minimum CL distance from the breast tangent. Contralateral lung maximum distance (CoLM) was defined analogously as the largest CL distance from the tangent.

Two parameters depicted the position of the contralateral breast. Minimum breast distance (MBD) was taken as the distance of the contralateral breast from the breast tangent. Breast-to-breast distance (BBD) was measured as the minimum distance between the breasts in the midplane.

Intended primarily to capture the heart anatomy relative to the treated breast, maximum heart distance (MHD) was defined as the maximum extent of the heart beyond the breast tangent. If no part of the heart extended beyond the tangent, as is the case in Fig. 1 and in general for virtually all right-sided breast-cancer patients, the value of MHD was negative and corresponded to the distance of the heart from the tangent. Maximum heart length (MHL, not depicted in Fig. 1) was defined in cranio-caudal direction as the length of the heart part beyond the tangent. If no part of the heart extended beyond the tangent, especially for right-sided breast cancer, we scored simply MHL = 0, as contrary to MHD there is no clear way how to define negative MHL values. Heart contour height (HCH, not shown in Fig. 1) was measured as the length of the whole heart, in the direction parallel to leaf opening of the collimator.

Finally, as additional measures of the thorax anatomy, rib and sternum chord lengths (RCL, SCL) were defined as corresponding segments of the breast tangent. Thorax curvature (ThC) was defined in the midplane as the angle between the breast tangent and a tangent to the rib cage, as illustrated in Fig. 1.

In this work, a subset of 2–3 out of these 17 parameters was searched for that should reflect anatomy-dependent variability in mean doses and further dose-volume metrics of the lung, as described below.

### Data analysis

Dose-volume histograms were generated for each patient and each technique, separately for IL and CL. Mean doses and further dose-volume metrics such as volume fraction V_40 Gy_ receiving dose above 40 Gy or dose D_10%_ to the most exposed 10% of the organ were extracted. The motivation to consider not only mean doses but also further dose-volume metrics was twofold. First, there are indications that radiation-induced pneumonitis, or generally lung damage, is likely related to high-exposed lung parts^19,20^. Second, the long-term risk of radiation-induced lung cancer likely deviates from a linear dose–response relationship, with enhanced contribution from low doses^21,22^.

Following our previous work on doses to contralateral breast^16^, the possibility to cover the inter-individual variability in diverse dose-volume metrics of the lung with the help of anatomic features was tested by fitting the data with generalized linear models^23^ (GLM) with logit link functions. Logit link functions are of sigmoidal shape. Contrary to commonly used linear models, GLM with logit link functions automatically possess both lower and upper constraints, as the dose-volume metrics do. Apart from trivial constraints (non-negative doses and 0–100% volume fractions), in this work an upper limit D_m_ at 110% of the prescribed whole-breast dose was taken, since hot spots in ipsilateral lung close to this value were found in a few plans. The TPS-generated dose-volume metrics were fitted by

1 $${\text{D}} = {\text{D}}_{{\text{m}}} /\left( {{1} + {\text{exp}}\left( { - \beta_{0} - \Sigma_{{\text{i}}} \beta_{{\text{i}}} {\text{X}}_{{\text{i}}} } \right)} \right),{\text{ V}} = {1}/\left( {{1} + {\text{exp}}\left( { - \beta_{0} - \Sigma_{{\text{i}}} \beta_{{\text{i}}} {\text{X}}_{{\text{i}}} } \right)} \right).$$

Here the linear predictor β_0_ + Σ_i_β_i_X_i_ with model coefficients β may be univariate (e.g. X_1_ = CLD) or include a linear combination of several anatomic features (e.g. X_1_ = MLW, X_2_ = CLD and X_3_ = MHD).

It is convenient to use dose-volume metrics D_0_ = D_m_/(1 + exp(− β_0_)), V_0_ = 1/(1 + exp(− β_0_)) at the hypothetical extrapolated case of X = 0 (e.g. MLW = CLD = MHD = 0) instead of the intercepts β_0_. Then the logit function can be approximated by an exponential expression valid especially at low doses or small volume fractions (D << D_m_ or V << 1),

2 $${\text{D}} \approx {\text{D}}_{0} {\text{exp}}\left( {\Sigma_{{\text{i}}} \beta_{{\text{i}}} {\text{X}}_{{\text{i}}} } \right),{\text{ V}} \approx {\text{V}}_{0} {\text{exp}}\left( {\Sigma_{{\text{i}}} \beta_{{\text{i}}} {\text{X}}_{{\text{i}}} } \right).$$

In this work, separately for each dose-volume metric studied, each combination of up to 3 out of the 17 scored anatomic features was tested as a predictor set using a dedicated GLM model. Testing all parameter combinations was found superior to a stepwise method that gradually added a single parameter best improving the model performance, since the stepwise method may occasionally miss the optimal combination for correlated predictors, as is the case here (Supplementary Material). The fitting was performed in Matlab (The MathWorks Inc., Natick, MA, USA). Model performance was assessed by fraction of variability in the data explained. To guide the model selection process, the Akaike information criterion (AIC) was used, which accounts for both the goodness of fit and the simplicity of the model. Prediction uncertainties of the models were calculated as described in the Supplementary Material.

### Ethics approval and consent to participate

This analysis was done according to the standards of the Helsinki Declaration and with permissions from the Ethics Committee of the Medical Faculty of the University of Rostock. Written informed consent to use and publish the data was obtained from the patients. All data used in this study was anonymized before import.

## Results

Whole-breast irradiation leads to a considerable dose burden to the IL. The mean value and standard deviation of TPS-calculated mean IL doses over all tangential plans, i.e. all studied patients and multiple plans per patient, amounted to (7.2 ± 1.7) Gy, which is almost 15% of the whole-breast prescribed dose of 50.4 Gy. Mean CL doses were much lower, (0.52 ± 0.16) Gy, i.e. about 1% of the prescribed dose. The values of mean IL and CL doses for individual tangential techniques and for multi-field IMRT are listed in Table 1, separately for left- and right-sided breast cancers as well as for the two contributing centres. For IL, the differences between centres, lateralities and techniques are generally smaller than the individual variability. Mean IL doses from 3DCRT-w and FFF were comparable (p > 0.3 from Wilcoxon signed rank test), while both 3DCRT+w and FF resulted in higher doses (p < 0.005). Multi-field IMRT resulted in notably higher IL doses than 3DCRT-w (p < 0.001). The calculated CL doses differed between the centres, increased upon the use of wedges (p < 0.001) or flattening filter (p < 0.001) and even more with multi-field IMRT (p < 0.001), but the dose levels remained low compared to IL doses.

**Table 1:** Mean doses to the ipsilateral and contralateral lung (IL, CL) for patients with left- or right-sided breast cancer.

Centre	Technique	Mean IL dose (Gy)		Mean CL dose (Gy)	
		Left	Right	Left	Right
1	3DCRT-w	7.2 ± 1.8	6.9 ± 1.6	0.47 ± 0.07	0.42 ± 0.07
	3DCRT+w	7.6 ± 1.9	7.2 ± 1.5	0.73 ± 0.09	0.68 ± 0.10
	FF	7.4 ± 1.8	7.1 ± 1.5	0.47 ± 0.07	0.42 ± 0.07
	FFF	7.3 ± 1.8	6.9 ± 1.5	0.37 ± 0.06	0.33 ± 0.06
2	3DCRT-w	6.5 ± 1.5	7.9 ± 1.4	0.73 ± 0.10	0.69 ± 0.10
	IMRT	8.0 ± 2.0	10.3 ± 1.9	1.13 ± 0.48	1.26 ± 0.55

Listed are the average values and standard deviations over the respective patient groups.

**Figure 2:**
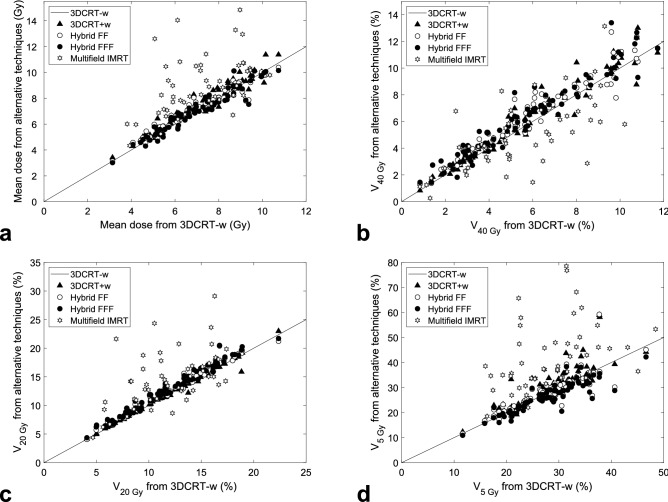
Lung exposure from alternative techniques of whole-breast irradiation with prescribed dose of 50.4 Gy. Displayed are (**a**) mean doses, (**b**) volume fractions V_40 Gy_, (**c**) V_20 Gy_ and (**d**) V_5 Gy_ for ipsilateral lung, as calculated by the TPS. Data from 3DCRT with wedges (triangles), hybrid technique with a flattening filter (empty circles), hybrid flattening filter-free technique (full circles) and multi-field IMRT (hexagrams) are plotted against those for 3DCRT without wedges for the same patient. Points above (or below) the identity line mean that the given technique resulted in a higher (or lower) dose than 3DCRT-w.

The variability in lung exposures between individual patients and between techniques is illustrated in more detail in Fig. 2. For some patients the mean IL doses from all techniques were as low as 3–5 Gy, while for others they exceeded 10 Gy (Fig. 2a). Even larger was the variability seen in high-exposed IL parts. For instance, the volume fraction V_40 Gy_ ranged from 1 to 13% (Fig. 2b). For many patients, V_40 Gy_ from IMRT were considerably lower than from tangential techniques. However, this advantage of IMRT in reducing the high-exposed volume vanished already for V_20 Gy_ (Fig. 2c). Importantly, this advantage of IMRT came at the expense of increased volumes exposed to lower doses. This can be seen in V_5 Gy_ (Fig. 2d) or in mean CL doses (Supplementary Figure S3). Also these dose-volume metrics exhibited a high variability between patients. Differences among diverse tangential techniques were rather limited, except for a clear separation of these techniques at low doses (Supplementary Figures S2–S3).

**Figure 3:**
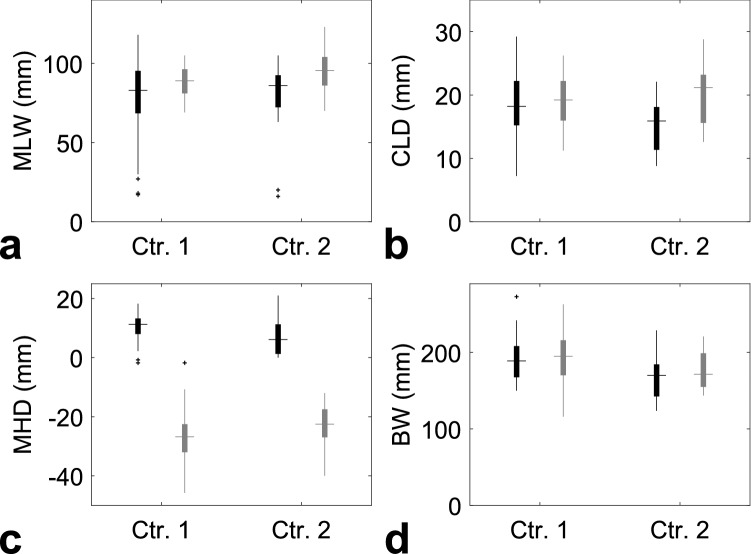
Individual variability of anatomic features that were found most influential on lung doses. Boxplots indicate the distribution of these measures among respective patient groups, separately for centres 1 and 2 and also for patients with left- and right-sided breast cancer (in black and grey). The mid line shows the median of the group, the box covers its 25th to 75th percentile, the whiskers denote the range of values not considered outliers, and the points show these outliers.

The extent of anatomic variability among patients is illustrated in Fig. 3. Shown is the variability in four features found to be most influential upon lung dose-volume metrics (as discussed below), namely central lung distance (CLD), midplane lung width (MLW), maximum heart distance (MHD) and breast width (BW). The distributions of patients’ anatomic parameters in the two centres largely overlapped. Only minor differences were seen between the two lateralities, except for MHD which was positive for left- and negative for right-sided breast-cancer patients since typically a part of the heart was included in the fields for left-sided cases but the heart was well separated from the fields for the other laterality.

**Figure 4:**
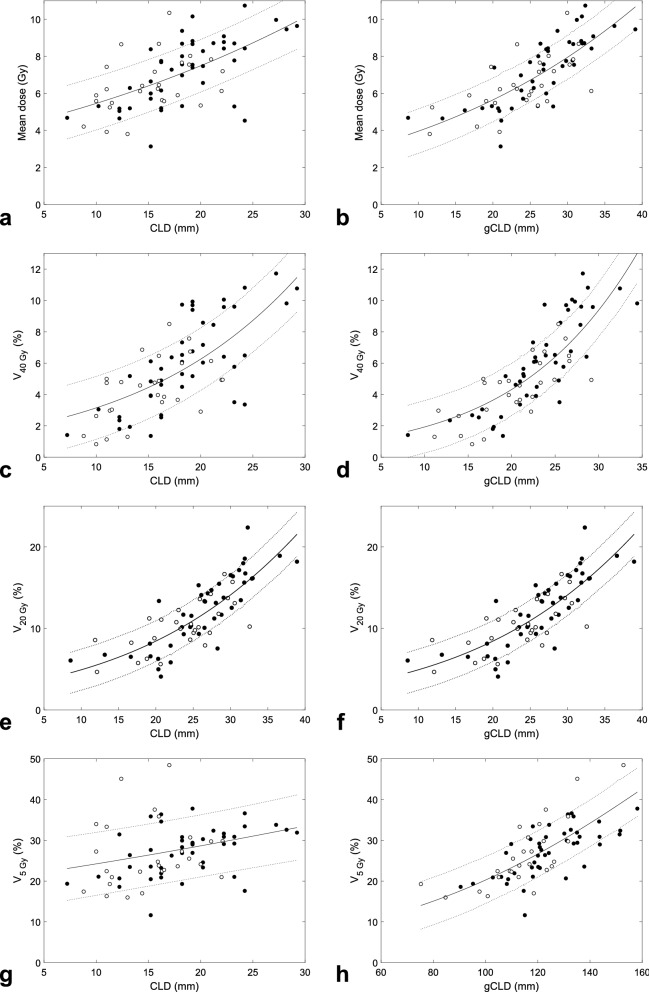
Linking dose-volume metrics to anatomic features: data for individual patients with left-sided breast cancer irradiated with 3DCRT without wedges in centres 1 and 2 (filled and empty circles) and the corresponding logit GLM fits (solid lines) with their prediction uncertainties (dotted lines). Mean doses to the ipsilateral lung (**a**,**b**) and volume fractions exposed to 40, 20 or 5 Gy (**c**–**h**) can be related to central lung distance (CLD, **a**,**c**,**e**,**g**) or to a linear combination of 2–3 anatomic features (**b**,**d**,**f**,**h**) listed in Table 2. To facilitate comparison with CLD-based models, the given linear combinations are presented in terms of ‘generalized CLD’, given by gCLD = Σ_i_(β_i_/Σ_j_β_j_)X_i_; for CLD-based models, gCLD = CLD, hence its name. For mean dose to the ipsilateral lung (**b**) gCLD = 0.17 MLW + 0.53 CLD + 0.30 MHD, for V_40 Gy_ (**d**) gCLD = 0.12 MLW + 0.58 CLD + 0.30 MHD, for V_20 Gy_ (**f**) gCLD = 0.16 MLW + 0.62 CLD + 0.22 MHD, while for V_5 Gy_ (**h**) gCLD = 0.59 MLW + 0.41 BW. The prediction uncertainties (Supplementary Material) are shown as the prediction plus/minus one standard deviation, i.e. the given dose-volume metric should be found within this prediction interval for 68% of new patients.

As suggested previously^6–9^, inter-patient variations in central lung distance (CLD) partly explained the individual variability in diverse lung dose-volume characteristics. For instance, among left-sided breast-cancer patients treated with 3DCRT-w, CLD-based GLM models (listed in the left-hand side of Table 2 and graphically presented in Fig. 4a,c,e,g) explained the individual variability in IL mean doses to 36%, in V_40 Gy_ to 48%, in V_20 Gy_ to 45%, but in the low-dose metric V_5 Gy_ only to about 8%.

**Table 2:** Summary of GLM models (Eq. (1)) and fraction of individual variability explained for selected dose-volume characteristics of the ipsilateral lung resulting from whole-breast irradiation using 3DCRT without wedges in left-sided patients.

Dose-volume metric	CLD-based models			MLW, CLD, MHD- or MLW, BW-based models					
	D_0_ (Gy) or V_0_ (%)	β_CLD_ (cm^−1^)	Expl. var. (%)	D_0_ (Gy) or V_0_ (%)	β_MLW_ (cm^−1^)	β_CLD_ (cm^−1^)	β_MHD_ (cm^−1^)	β_BW_ (cm^−1^)	Expl. var. (%)
D_mean_	3.93	0.358 ± 0.057	36	2.75	0.068 ± 0.013	0.205 ± 0.055	0.116 ± 0.044		58
V_40 Gy_	1.56	0.719 ± 0.091	48	0.85	0.099 ± 0.022	0.483 ± 0.086	0.248 ± 0.073		66
V_20 Gy_	4.77	0.540 ± 0.072	45	2.83	0.092 ± 0.017	0.355 ± 0.068	0.129 ± 0.055		65
V_5 Gy_	20.3	0.228 ± 0.095	8	4.07	0.106 ± 0.017			0.073 ± 0.012	50

Model coefficients are reported as best estimates and their standard errors.

To improve these figures, alternative combinations of anatomic features (for practical reasons up to three features only) were tested as predictors of lung dose-volume metrics. CLD complemented by MLW and MHD was identified as the triplet that best explained the individual variability in mean IL doses. For patients with left-sided breast cancer, the individual variability in mean IL dose from 3DCRT-w was explained to 58% (Table 2, Fig. 4b), notably better than the above-mentioned figure of 36% for CLD alone; correspondingly, the AIC criterion decreased by 26.5 points, quantifying the superiority of the triplet-based model. The full (Eq. (1)) and approximate forms (Eq. (2)) of the given GLM model read

$$\begin{aligned} {\text{D}}_{{{\text{IL}}}} \left[ {{\text{Gy}}} \right] = & {1}.{1} \times {5}0.{4}/\left( {{1} + {\text{exp}}\left( { - \left( { - {2}.{95} + 0.0{\text{68 MLW}}\left[ {{\text{cm}}} \right] + 0.{2}0{\text{5 CLD}}\left[ {{\text{cm}}} \right] + 0.{\text{116 MHD}}\left[ {{\text{cm}}} \right]} \right)} \right)} \right) \\ \approx & { 2}.{\text{75 exp}}\left( {0.0{\text{68 MLW}}\left[ {{\text{cm}}} \right] + 0.{2}0{\text{5 CLD}}\left[ {{\text{cm}}} \right] + 0.{\text{116 MHD}}\left[ {{\text{cm}}} \right]} \right). \\ \end{aligned}$$

The predicted mean IL dose thus increases by about 7%, 23% and 12% per 1 cm increase in MLW, CLD and MHD, respectively.

Also the models for V_40 Gy_ and V_20 Gy_ were improved upon adding MLW and MHD as compared with CLD alone (Fig. 4, Table 2). At medium and low doses, MLW and BW turned out to be influential, explaining e.g. for V_5 Gy_ the individual variability to 50% (Fig. 4h, Table 2).

As presented in the Supplementary Tables S2–S3, similar results were obtained also for further dose-volume metrics, for right-sided breast cancer, and also for other tangential techniques. Actually, since lung doses from alternative tangential techniques are similar to those from 3DCRT-w (Fig. 2), the models presented here can be used not only for 3DCRT-w, but as an approximation also for 3DCRT+w as well as for hybrid FF and FFF techniques. However, lung doses from multi-field IMRT were only weakly correlated with the studied anatomic features.

## Discussion

TPS data were gathered for 128 breast-cancer patients planned for treatment in two centres using multiple irradiation techniques. The presented lung doses are within the ranges reported from contemporary breast-cancer radiotherapy^10^. They highlight the large inter-patient variability. To explain this variability, alternative combinations of numerous anatomic measures recorded within the PASSOS project were tested as predictors of patient-specific lung doses. For practical reasons, only up to 3 parameters were considered; including additional parameters or allowing interaction or quadratic terms improved the explained variability and the AIC criterion only marginally.

CLD has been suggested previously^6–9^ as a useful anatomic parameter that correlates with IL doses. As shown here, this holds also for contemporary tangential techniques of 3D-conformal breast-cancer radiotherapy, but CLD captures the individual variability in mean IL doses to only about 40%, leaving about 60% unexplained. Complementing CLD by MLW and MHD, the opposite holds: the individual variability is explained to about 60%, leaving only about 40% unexplained. Similar to CLD alone, also this triplet works especially in the high-dose region. CLD and MLW represent two complementary, perpendicular measures of the extent of the ipsilateral lung covered by the tangential fields (Fig. 1). Hence, their involvement as predictors especially for high-dose regions is obvious. It is fortunate that the third anatomic measure relevant with respect to lung doses is MHD, a well-known parameter that largely determines doses to the heart^9,24^; this reduces the number of anatomic measures needed to capture the variability in overall long-term risks, which are largely driven by exposures of the lung, heart and contralateral breast^17,18^. Finally, BW affects lung parts exposed to low doses presumably since it is linked with the amount of scatter from the treated breast.

Interestingly, in spite of the heart and lung asymmetry, the mean doses and high-dose metrics were largely similar for left- and right-sided breast-cancer patients (Table 2; Supplementary Tables S2-S3). The same parameters were found influential; note that MHD was extended to negative values when the heart was located outside the tangential fields, depicting then the heart distance (separation) from the breast tangent rather than the heart part within the field. Also the dependence of mean doses and high-dose metrics on anatomic parameters was similar for left- and right-sided cases.

However, differences between the two participating centres were observed. At high doses these differences were relatively small. They corresponded to differences in anatomic features between the patient groups: patients with left-sided tumours in centre 1 possessed larger CLD and MHD (Fig. 3) and hence received larger ipsilateral lung doses than those in centre 2, while the opposite was the case for patients with right-sided tumours. At low doses, e.g. for mean CL doses, the inter-centre differences could be largely traced back to the well-known inaccuracy of TPS calculations at low doses^25^, since the TPS tended^15,16^ to underestimate low doses in centre 1 and overestimate them in centre 2.

Another source of differences between the centres is the inter-observer variability in contouring and plan approval, despite using the RTOG guidelines. Within the PASSOS project, we have especially looked into this issue for the heart^26^. However, we have intentionally not forced the two centres to come to a common procedure but let them contour, perform the planning and accept plans as they are used to. This for sure led to some differences, both in the plans and in the scored anatomic features. Likely it affected the resulting anatomy-dependent models too; models adjusted to datasets from a single centre performed better than the presented ones that are a kind of compromise between centre-specific ones. On the other hand, by allowing for some inter-centre and inter-observer variability, the obtained results are more robust and more relevant for a new centre, and prediction bands reflect the underlying uncertainty more appropriately.

The reported results will be used as anatomy-dependent estimates of lung doses within the PASSOS software for personalized assessment of long-term health risks following breast-cancer radiotherapy, if the user does not provide dose-volume characteristics from the actual treatment plan. The reported database of anatomy-dependent lung doses from tangential breast-cancer radiotherapy and the resulting models can also be used to quickly identify favourable as well as problematic anatomies. Fulfilling dose-volume constraints on e.g. mean lung dose, V_20 Gy_ and V_30 Gy_ (cf. Refs.^27–29^) may be challenging for highly problematic anatomies. For favourable ones, this is quite easy; however, stopping the optimization process as soon as these generic constraints have been met would be suboptimal. There is likely room for improvement if a calculated plan provides larger lung doses than the reported ones at the given anatomy. The present results thus may serve as a fast benchmark for judging the quality of treatment plans.

The results may also support efficient use of techniques such as multi-field IMRT or volumetric modulated arc therapy (VMAT). First, the results enable one to quickly compare such plans with lung doses achievable with tangential techniques. Second, patients can be identified for whom large lung parts would be exposed to high doses with tangential techniques and for whom IMRT or VMAT may largely reduce the risk of complications such as pneumonitis, which may be beneficial despite coming at the cost of enhanced low-dose wash. For instance, the results presented in Fig. 2 show that when 3DCRT yields V_40 Gy_ higher than about 6%, the size of this high-exposed part can often be largely reduced by intensity-modulated techniques. This high V_40 Gy_ occurs typically for left-sided patients for whom 0.12 MLW + 0.58 CLD + 0.30 MHD > 25 mm (Fig. 4). Therefore, especially patients with such anatomies may benefit from IMRT or VMAT. Alternatively, other techniques that reduce lung exposures may be employed for these patients, such as deep-inspiration breath hold or prone or lateral decubitus positioning^10,21,30^.

The present analysis has been limited to whole-breast irradiation, and focused on classical tangential irradiation techniques. To enhance its clinical relevance, future work shall extend these results to VMAT treatment plans, partial breast irradiation as well as plans with RNI. To facilitate clinical applications, the results will be combined with low- and high-dose evidence-based risk models^31^ and implemented into a dedicated risk assessment software package.

## Conclusion

Differences in patient anatomy lead to large variations in lung doses from whole-breast irradiation, for instance with mean doses to the ipsilateral lung ranging from 3 to 11 Gy for tangential techniques. The majority of the inter-individual variability in mean doses and further dose-volume metrics can be explained by a few anatomic features, namely MLW, CLD and MHD for high-dose metrics, and MLW and BW for low-dose metrics. The resulting models will be implemented in the PASSOS software to provide anatomy-dependent estimates of lung doses as is necessary for assessing personalized long-term health risks from breast-cancer radiotherapy. Using the present results, criteria can be defined to easily identify patients with problematic anatomies who would benefit from using irradiation techniques such as multi-field IMRT or VMAT to reduce lung volumes exposed to high doses. The results also point to patients with favourable anatomies who could safely be treated with tangential techniques that lead to reduced low-dose burden and mean doses to diverse organ at risks and consequently lower long-term cardiac and secondary cancer risks than multi-field techniques.

## Supplementary Information

Supplementary Information.

## Data Availability

The datasets generated and analysed during the current study are available from the corresponding author on reasonable request.
