# Supplementary material for: Anatomy-dependent lung doses from 3D-conformal breast-cancer radiotherapy
Source: Sci Rep. 2022 Jun 28;12:10909. doi: 10.1038/s41598-022-14149-2 (PMC9240052; doi:10.1038/s41598-022-14149-2)
Supplement: Supplementary file 1 — Supplementary Information. [file 41598_2022_14149_MOESM1_ESM.pdf]

Supplementary Material

Anatomy-dependent lung doses from 3D-conformal breast-cancer radiotherapy

Pavel Kundrát [a, b, 1], Hannes Rennau [c], Julia Remmele [d], Sabine Sebb [c], Cristoforo Simonetto [a], Jan Christian Kaiser [a], Guido Hildebrandt [c], Ulrich Wolf [d], Markus Eidemüller [a]

[a] Institute of Radiation Medicine, Department of Radiation Sciences, Helmholtz Zentrum München – German Research Center for Environmental Health (GmbH), Neuherberg, Germany
[b] Department of Radiation Dosimetry, Nuclear Physics Institute of the CAS, Prague, Czech Republic
[c] Department of Radiation Oncology, University of Rostock, Rostock, Germany
[d] Department of Radiation Oncology, Leipzig University, Leipzig, Germany

[1] **Corresponding author;** pavel.kundrat@helmholtz-muenchen.de, kundrat@ujf.cas.cz

Correlations of anatomic measures

The anatomic measures scored in the present work are not fully independent but mutually correlated. In Figure S1, correlation coefficients between pairs of these measures are presented for the given patient set. In particular, closely correlated are the breast-to-breast distance BBD with the minimum breast distance MBD, or thorax curvature ThC with either rib chord length RCL or maximum lung distance MLD. For patients with right-sided breast cancer, MHL = 0, and hence this feature was omitted from the figure.

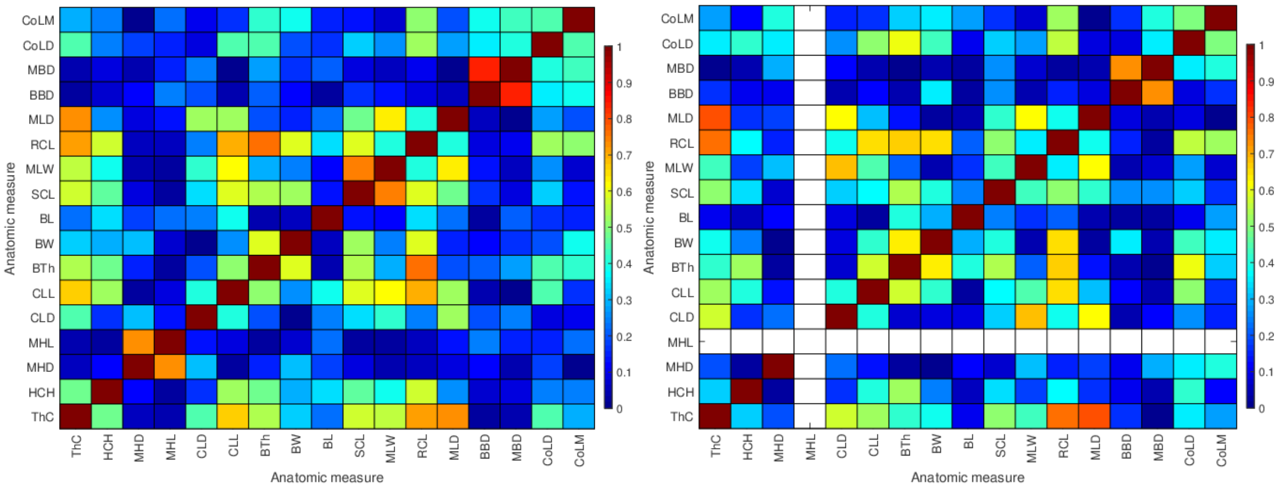

Figure S1: Pearson's correlation coefficients between pairs of anatomic measures for patients with left- or right-sided breast cancer (left and right panel, respectively).

Dose-volume metrics of the lung and their dependence on anatomic features

In addition to the results presented in the main text, further dose-volume metrics of the lung following alternative whole-breast irradiations are presented in Figure S2, namely volume fractions $V_{10\,Gy}$, $V_{2\,Gy}$, and $V_{1\,Gy}$. All these metrics show a high inter-patient variability. $V_{10\,Gy}$ from tangential techniques are markedly lower than those from IMRT. The same holds also at dose levels of 2 Gy and 1 Gy. At these rather low doses, different tangential techniques clearly separate, since the additional scatter introduced by wedges or flattening filters becomes important. This separation of diverse tangential techniques can be seen also for mean CL doses presented in Figure S2. The separation of data between the two centres largely follows from inaccuracy of TPS calculations, as discussed in the main text.

To illustrate the roles of individual anatomic features, the fractions of inter-patient variability in mean doses to the ipsilateral lung that can be explained with single anatomic features are listed in Table S1. MLW, CLD and MLD belong to the most influential single parameters for both left- and right-sided cases. However, other parameter combinations than this triplet may be better suited as multivariate model predictors, as these particular parameters are mutually rather correlated (Figure S1). For instance, upon selecting CLD into the predictor set, the variability left unexplained will likely be best covered by parameters rather uncorrelated with CLD. Adding further parameters in a stepwise manner would address this issue. However, in detailed tests using the present database, we have seen that occasionally the stepwise procedure missed the best parameter set. Given the relatively limited number of parameters scored, we have thus tested all parameter combinations (up to triplets) as described in the Methods.

To complement Table 2, anatomy-dependent models are presented in Tables S2 and S3 for diverse dose-volume metrics of the lung, using CLD alone, the triplet CLD, MLW and MHD, or the pair MLW and BW.

Hybrid FF and FFF techniques as well as 3DCRT+w were planned for the patients in centre 1 only, while 3DCRT-w was the standard technique calculated for all patients in both centres. Dedicated models for alternative variants of tangential techniques could thus be derived for centre 1 only. However, lung doses from alternative tangential techniques are very similar, at least at high doses (Figures 2 and S2). Models and coefficients derived for 3DCRT-w (Tables 2 and S2-3) thus largely capture the individual variability in lung doses also for other tangential techniques, for instance for mean IL doses from 3DCRT+w, FF and FFF to 57, 60 and 63% for left-sided and to 56, 60 and 59% for right-sided breast-cancer patients, respectively (not shown). In the region of relatively low doses, systematic differences between alternative tangential techniques occur (Figures S2-S3), so that technique-specific parameters would be needed in the corresponding models (not shown).

The individual variability in lung doses from IMRT is largely unrelated to the studied anatomic features. For right-sided cases, MLD covered 27% of individual variability in mean IL doses. For left-sided cases, however, no parameter was significant at $p < 0.05$ (not shown). However, these results may be biased by having planned IMRT only for 27 patients with left-sided and 23 with right-sided breast cancer.

Since CL doses are relatively low, they possess rather low absolute variability as well. Capturing this variability is, hence, not as critical as for the IL. Yet, the anatomic features studied in this work can be used for this purpose too. Among patients with left-sided breast cancer, for instance, CoLD and BW together captured 33% of the variability in mean CL doses and 40% in $D_{10\%}$ for CL, while CoLM covered 28% of the variability in $D_{90\%}$ for CL (not shown).

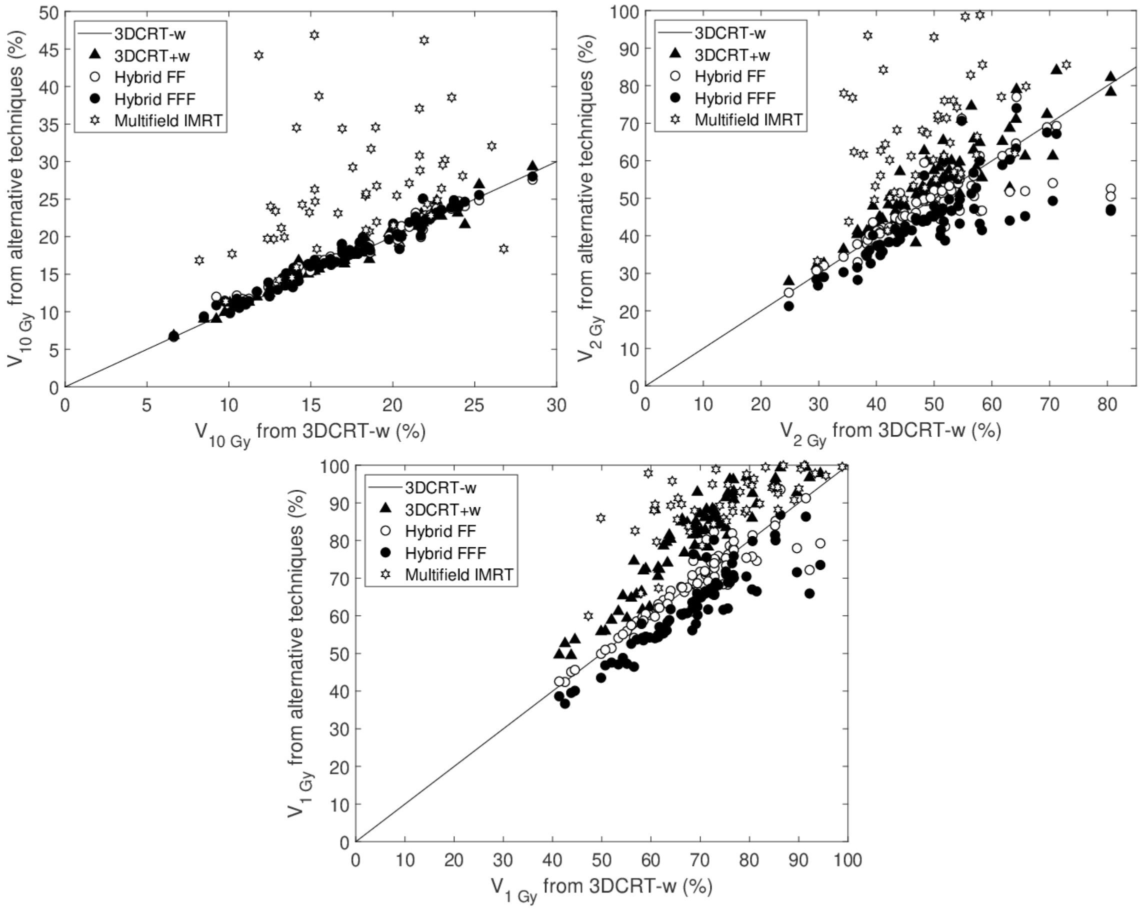

Figure S2: Volume fractions of ipsilateral lung receiving 10, 2 or 1 Gy from alternative whole-breast irradiation techniques. Displayed are mean doses to contralateral lung in individual patients, as calculated by the TPS. Data from 3DCRT with wedges (triangles), hybrid technique with a flattening filter (empty circles), hybrid flattening filter-free technique (full circles) and multifield IMRT (hexagrams) are plotted against those for 3DCRT without wedges for the same patient. Points above (or below) the identity line mean that the given technique resulted in a higher (or lower) dose than 3DCRT-w.

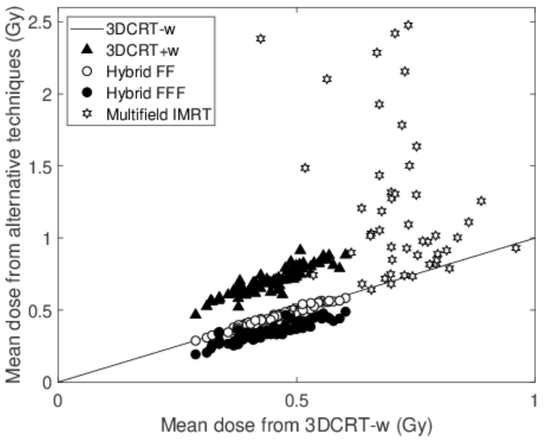

Figure S3: Mean doses to the contralateral lung from alternative techniques of whole-breast irradiation with prescribed dose of 50.4 Gy. Shown are TPS-calculated mean doses to contralateral lung in individual patients. As in Figure S2, data from alternative techniques (symbols) are plotted against those for 3DCRT without wedges for the same patient. In the shown low-dose region, actual doses tend to be underestimated by the TPS in centre 1 and overestimated in centre 2, hence the systematic shift of IMRT data to the right.

Table S1: Fraction of inter-patient variability in mean dose to the ipsilateral lung explained by a GLM using a single anatomic feature as predictor. N.S.: parameter not statistically significant, hence not included in the model and not explaining any individual variability at all.

Anatomic feature	Explained variability (%)	
	Left-sided cases	Right-sided cases
BW	N.S.	N.S.
BTh	N.S.	N.S.
BL	11	N.S.
MLW	39	45
MLD	40	25
CLD	36	38
CLL	28	14
CoLD	N.S.	N.S.
CoLM	N.S.	N.S.
MBD	N.S.	N.S.
BBD	N.S.	N.S.
MHD	10	20
MHL	N.S.	N.S.
HCH	N.S.	N.S.
RCL	N.S.	N.S.
SCL	13	N.S.
ThC	25	10

Table S2: Summary of GLM models (Eq.1) and fraction of individual variability explained for dose-volume characteristics of the ipsilateral lung resulting from whole-breast irradiation with 3DCRT without wedges in left-sided breast-cancer patients. Model coefficients are reported as best estimates and their standard errors. N.S.: parameter not statistically significant, hence not included in the model.

	CLD-based models			MLW, CLD, MHD- or MLW, BW-based models					
Dose-volume metric	D_0 (Gy) or V_0 (%)	β_{CLD} (cm^{-1})	Expl. var. (%)	D_0 (Gy) or V_0 (%)	β_{MLW} (cm^{-1})	β_{CLD} (cm^{-1})	β_{MHD} (cm^{-1})	β_{BW} (cm^{-1})	Expl. var. (%)
D_{mean}	3.93	0.358 ± 0.057	36	2.75	0.068 ± 0.013	0.205 ± 0.055	0.116 ± 0.044		58
$D_{1\%}$	39.9	0.340 ± 0.052	38	37.9	0.032 ± 0.010	0.210 ± 0.054	0.158 ± 0.043		52
$D_{2\%}$	35.8	0.431 ± 0.066	38	32.7	0.048 ± 0.012	0.249 ± 0.066	0.202 ± 0.052		54
$D_{5\%}$	19.9	0.796 ± 0.137	34	13.1	0.127 ± 0.023	0.389 ± 0.126	0.331 ± 0.101		56
$D_{10\%}$	5.57	1.146 ± 0.178	42	1.76	0.199 ± 0.036	0.748 ± 0.162	0.345 ± 0.122		63
$D_{20\%}$	2.79	0.689 ± 0.108	36	1.63	0.101 ± 0.028	0.541 ± 0.107	N.S.		48
$D_{30\%}$	2.88	0.269 ± 0.091	11	0.66	0.100 ± 0.018			0.064 ± 0.011	45
$D_{40\%}$				0.34	0.088 ± 0.017			0.079 ± 0.012	47
$D_{50\%}$				0.19	0.091 ± 0.019			0.089 ± 0.013	45
$V_{40\ Gy}$	1.56	0.719 ± 0.091	48	0.85	0.099 ± 0.022	0.483 ± 0.086	0.248 ± 0.073		66
$V_{30\ Gy}$	3.15	0.624 ± 0.076	49	1.80	0.095 ± 0.018	0.429 ± 0.072	0.162 ± 0.059		67
$V_{20\ Gy}$	4.77	0.540 ± 0.072	45	2.83	0.092 ± 0.017	0.355 ± 0.068	0.129 ± 0.055		65
$V_{10\ Gy}$	9.25	0.394 ± 0.075	29	6.09	0.087 ± 0.017	0.249 ± 0.069	N.S.		50
$V_{5\ Gy}$	20.3	0.228 ± 0.095	8	4.07	0.106 ± 0.017			0.073 ± 0.012	50
$V_{2\ Gy}$				8.70	0.086 ± 0.017			0.086 ± 0.013	47

Table S3: Summary of GLM models (Eq.1) and fraction of individual variability explained for dose-volume characteristics of the ipsilateral lung resulting from whole-breast irradiation with 3DCRT without wedges in right-sided breast-cancer patients. Model coefficients are reported as best estimates and their standard errors. N.S.: parameter not statistically significant, hence not included in the model.

	CLD-based models			MLW, CLD, MHD- or MLW, BW-based models					
Dose-volume metric	D_0 (Gy) or V_0 (%)	β_{CLD} (cm^{-1})	Expl. var. (%)	D_0 (Gy) or V_0 (%)	β_{MLW} (cm^{-1})	β_{CLD} (cm^{-1})	β_{MHD} (cm^{-1})	β_{BW} (cm^{-1})	Expl. var. (%)
D_{mean}	3.83	0.359 ± 0.066	38	3.44	0.075 ± 0.023	0.176 ± 0.071	0.083 ± 0.029		57
$D_{1\%}$	36.2	0.460 ± 0.054	54	36.7	0.073 ± 0.015	0.212 ± 0.060	0.083 ± 0.025		75
$D_{2\%}$	29.4	0.640 ± 0.068	59	27.9	0.111 ± 0.018	0.281 ± 0.070	0.073 ± 0.030		80
$D_{5\%}$	15.3	0.964 ± 0.110	61	8.87	0.158 ± 0.038	0.587 ± 0.125	N.S.		71
$D_{10\%}$	5.89	1.055 ± 0.196	40	2.06	0.294 ± 0.071	0.552 ± 0.196	0.218 ± 0.086		62
$D_{20\%}$	3.60	0.505 ± 0.116	28	3.26	0.164 ± 0.035	N.S.	0.156 ± 0.052		46
$D_{30\%}$	2.76	0.300 ± 0.095	17	1.03	0.092 ± 0.027			0.041 ± 0.013	34
$D_{40\%}$	2.04	0.236 ± 0.110	8	0.66	0.058 ± 0.029			0.058 ± 0.014	31
$D_{50\%}$				0.68	N.S.			0.063 ± 0.016	24
$V_{40\ Gy}$	1.44	0.712 ± 0.124	43	1.19	0.136 ± 0.041	0.372 ± 0.123	0.148 ± 0.048		60
$V_{30\ Gy}$	3.30	0.562 ± 0.096	44	2.62	0.119 ± 0.032	0.277 ± 0.096	0.113 ± 0.039		62
$V_{20\ Gy}$	4.84	0.500 ± 0.087	42	3.80	0.114 ± 0.029	0.233 ± 0.088	0.100 ± 0.036		62
$V_{10\ Gy}$	8.62	0.413 ± 0.076	38	7.10	0.093 ± 0.027	0.195 ± 0.082	0.081 ± 0.034		56
$V_{5\ Gy}$	18.0	0.319 ± 0.091	20	7.58	0.092 ± 0.025			0.042 ± 0.012	36
$V_{2\ Gy}$	38.9	0.243 ± 0.115	8	13.6	0.071 ± 0.028			0.066 ± 0.015	34

Prediction uncertainty

Prediction intervals (i.e., confidence intervals on new observations) were estimated by generalizing the corresponding formulas for linear models to generalized linear models. In linear models, for a new datum x^{new} (new patient with anatomical parameters x_1^{new}, x_2^{new}, …), the model prediction y^{new} reads (in matrix notation, with ^{T} denoting transposition)

$$y^{new} = (1, x_1^{new}, x_2^{new}, \ldots)(\beta_0, \beta_1, \beta_2, \ldots)^T \quad \text{(S1)}$$

The confidence interval on the mean is

$$CI_{y\pm} = y^{new} \pm t\ (\quad x\ S\ x^T)^{1/2} \quad \text{(S2)}$$

and the prediction interval (which takes into account both the error from the fitted model and the error associated with future observations) reads

$$PI_{y\pm} = y^{new} \pm t\ (s^2 + x\ S\ x^T)^{1/2}. \quad \text{(S3)}$$

Here S is the estimated covariance matrix of the coefficient estimates, $S = (X^TX)^{-1}s^2$, where X is the design matrix, and $s^2 = SSE/DFE$ is the mean squared error (i.e., sum-of-squared error per degree of freedom). Factor t depends on the confidence level and DFE (given by the number of patients minus the number of coefficients in the model), and is computed using the inverse of Student's *t* cumulative distribution function.

For generalized linear models with logit link function applied to anatomy-dependent dose-volume metrics, three different scales are considered: x refers to anatomic parameters, y to their linear transformation (i.e., the predictor), and D to its logit transform (i.e., the given dose-volume metric). For a new datum x^{new} (patient's anatomic parameters), the predictor y^{new} and the model prediction D^{new} are given by

$$D^{new} = D_{max}/[1+\exp(-y^{new})] = D_{max}/[1+\exp[-(1, x_1^{new}, x_2^{new}, \ldots)(\beta_0, \beta_1, \beta_2, \ldots)^T]]. \quad \text{(S4)}$$

The confidence interval on the mean is obtained by logit transformation of $CI_{y\pm}$ for the linear model:

$$CI_{D\pm} = D_{max}/[1+\exp(-CI_{y\pm})] = D_{max}/[1+\exp(-[y^{new} \pm t\ (\quad x\ S\ x^T)^{1/2}])]. \quad \text{(S5)}$$

The residuals (on the dose scale) are approximately normally distributed. Thus the prediction interval was approximated in this work by quadratically adding CI_D and s similarly to the case of linear models,

$$PI_{D\pm} = D^{new} \pm [t^2\ s^2 + (CI_{D+} - D^{new})^2]^{1/2}. \quad \text{(S6)}$$

The standard deviation σ of this normal distribution (which includes 68.3% of the data) corresponds to t = 1 in the above formulas,

$$\sigma = [s^2 + (D_{max}/[1+\exp(-[y^{new} + (\quad x\ S\ x^T)^{1/2}])] - D^{new})^2]^{1/2}. \quad \text{(S7)}$$
